# Social Context, Self-Efficacy, and Patient-Centered Service Behavior of Medical Professionals: The Moderating Role of Achievement Motivation

**DOI:** 10.3389/fpsyt.2022.784228

**Published:** 2022-02-11

**Authors:** Xianhong Huang, Cong Wang, Le Hua, Yuan Gao, Siyu Zhou, Xiaohe Wang

**Affiliations:** ^1^Department of Health Policy and Management, School of Public Health, Hangzhou Normal University, Hangzhou, China; ^2^Affiliated Xixi Hospital, College of Medicine, Zhejiang University, Hangzhou, China

**Keywords:** social context, self-efficacy, achievement motivation, patient-centered, China

## Abstract

Patient-centered services are high-value, high-quality medical services that can improve patient satisfaction and safety. However, little is known about their driving mechanisms. This study examined whether external (social context) and internal (self-efficacy) motivation affects medical professionals' patient-centered service behavior, and explored the moderating role of medical professionals' achievement motivation. A cross-sectional survey was conducted with medical professionals at public hospitals in Hangzhou. Descriptive statistics, hierarchical linear regression analysis, and structural equation modeling were used to analyze the data. The final analysis included 1,612 medical professionals. Results indicated that perceived social context and self-efficacy had direct and positive effects (β = 0.578, β = 0.269) on medical professionals' patient-centered service behavior. Social context also indirectly influenced medical professionals' patient-centered service behavior mediated by self-efficacy (β = 0.149). Additionally, achievement motivation played a moderating role (β = −0.037) between the social context and self-efficacy. From the results it can be deduced that an excellent social environment and strong self-efficacy can promote the patient-centered service motivation of medical professionals. This promoting effect is even more significant under the moderating effect of high achievement motivation. Governments, health administrative departments, and hospital management should use internal and external motivation factors to promote medical professionals' patient-centered service behavior. Along with formulating relevant laws and regulations, efforts should also be made to guide medical professionals to improve their self-efficacy and achievement motivation, thereby encouraging patient-centered medical service behavior.

## Introduction

Patient-centered service is highly valued, as it helps in improving the quality of medical services and building a harmonious doctor–patient relationship (1). Balint first defined the concept of “patient-centered medicine” in 1955. He stated that medical professionals should understand the unique characteristics of the patient, their social context, and disease development during diagnosis and treatment of diseases (2). Many studies have confirmed that patient-centered services have positive effects on patient outcomes, such as improving patients' treatment compliance, reducing patients' risky behaviors, making the doctor–patient relationship more harmonious, and improving patients' health outcomes and satisfaction (3–5).

In the Chinese context, various techniques have been introduced to establish and consolidate the service concept of keeping the patient at the center of treatment. In 2009, the Opinions on Deepening the Reform of Medical and Health System, issued by the Central Committee of the Communist Party of China and the State Council (6), stated that it is necessary to establish a standardized operating procedure for public hospitals, follow the principles of public welfare and social benefits, promote patient-centered medical services, and improve patients' medical experiences. In 2016, the China Health and Family Planning Commission's action plan (7) suggested that continuous management of medical quality is necessary alongside disease diagnosis and treatment. Additionally, the importance of improving the service concept of “taking patients as the center and promoting multidisciplinary diagnosis and treatment mode” was emphasized. In the same year, the Report on Deepening the Reform of China's Medical and Health System (8), jointly issued by various organizations, including the World Bank Group, the World Health Organization, and China Health Planning Commission, also suggested that China should transform its healthcare system into a people- and quality-oriented integrated service delivery system, that is, establish a people-centered integrated care model. In 2018, China's National Health Commission and National Administration of Traditional Chinese Medicine formulated and issued the Action Plan for Further Improving Medical Services (2018–2020) (9). However, the concept of patient-centered services has not yet been adequately implemented in China. In 2017, a report showed that patients did not perceive the services as patient-centered (10). Further, the service provided by some medical professionals was unsatisfactory owing to their lack of an appropriate attitude and respect for patients' rights and interests. Furthermore, hospitals' focus on economic benefits and neglect of service quality has led to low management efficiency, imperfect supervision and improvement mechanisms, and high distrust toward medical professionals among patients. Therefore, clarifying the influencing factors and driving mechanisms of medical professionals' patient-centered service behavior can provide theoretical support to the government, health management departments and hospital managers when formulating policies and regulations.

Recently, scholars in China and other countries have studied the factors influencing medical professionals' patient-centered service behavior. For example, Dagmara et al. (11) explored the factors that promote and hinder the implementation of patient-centered services in medical institutions and personnel, and highlighted that creating good doctor–patient relationships, encouraging patients to participate in medical decision-making, and encouraging effective communication among medical personnel can promote patient-centered service behavior. Taylor and Groene (12) found that budget constraints, flawed policies and regulations, and a deficient work culture in the hospitals hinder the provision of patient-centered service. Waweru et al. (13) found that patient-centered services require joint efforts from doctors and patients.

However, the existing research has several shortcomings. First, most scholars have used qualitative research methods; thus, rendering insufficient quantitative evidence. Second, most existing research has simply screened influencing factors, and research on influencing mechanisms or paths is relatively lacking. Finally, regarding the influencing factors, most studies have focused on the social context in which medical professionals are located compared to the internal psychological factors. Although some studies have shown that patient-centered service behavior of medical professionals is influenced by external social and environmental factors and internal psychological factors (14), such studies are few. Therefore, this study explored how external social context and internal self-efficacy directly and indirectly influenced patient-centered service behavior of medical professionals, and further analyzed the moderating effect of achievement motivation on external social context and internal self-efficacy.

### Theoretical Basis

Motivation theory has been widely used in the fields of medical and health management (15, 16). It posits that motivation can stimulate behavior: positive motivation can promote good behavior, while negative motivation hinders it (3). In this study, patient-centered service of medical professionals was considered good behavior, driven by internal and external motives, and its driving mechanisms were worth exploring. Therefore, we could apply motivation theory to this study to explore the relationship between the internal and external motivations of medical professionals and their patient-centered service behavior. Internal motivation mainly refers to the self-efficacy of medical professionals (effective communication confidence and interpersonal communication confidence) (4). External motivation mainly refers to the support given to medical professionals in their social context (policies and regulations, social relations, and patient support) (5).

### Research Hypotheses

#### Relationship Between Social Context and Patient-Centered Service Behavior of Medical Professionals

**Figure 1:**
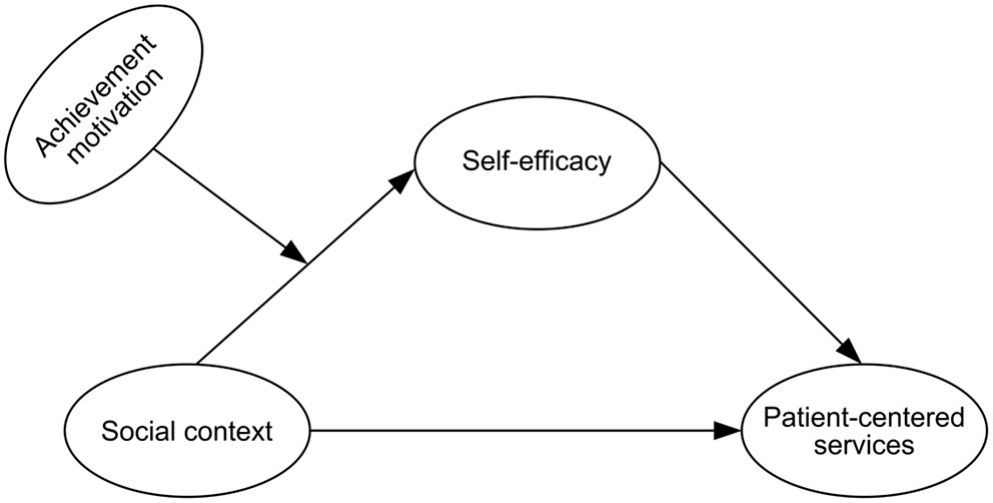
Research model diagram of medical professionals' patient-centered service behavior.

Figure 1 shows the impact of social context on medical professionals' patient-centered service behavior. Environmental conditions directly affect the formation and change of individual attitudes and behaviors. Taylor and Groene (12) found that economic constraints, budget constraints, health policies, and regulations are important factors that affect the patient-centered service of medical professionals. A qualitative study by Gross (17) showed that when medical professionals receive support from colleagues, they are more willing to share their feelings with others to enhance the interaction between medical teams, doctors, and patients; and to support patient participation in decision-making. Dagmara et al. (11) found that patients' positive attitudes, trust, respect, and social support promoted effective communication; and patients' socio-economic status also affected patient-centered communication.

In summary, environmental conditions change the working behavior and mood of medical professionals. Therefore, the following hypothesis was formulated.

**Hypothesis 1 (H**_**1**_**)**: A positive social context has a positive effect on the patient-centered service behavior of medical professionals.

#### Relationship Between Self-Efficacy and Patient-Centered Service Behavior of Medical Professionals

Self-efficacy refers to people's confidence in their ability to execute an activity with competence (18). Zheng (19) believed that self-efficacy is the internal driving factor of doctors' excellent performance. The higher the self-efficacy, the higher the motivation and enthusiasm for work, and the higher the work performance. Additionally, Li et al. (20) found that nursing students' self-efficacy was positively correlated with their cooperative attitude at work. Welsh et al. (21) reported that cultivating the self-efficacy of primary care doctors was helpful in improving doctor–patient communication skills. Self-efficacy seems to subjectively improve the efforts of medical professionals and play a positive role in their social relations, work attitude, work behavior, and work efficiency. Therefore, the following hypothesis was proposed.

**Hypothesis 2 (H**_**2**_**)**: Self-efficacy has a positive effect on the patient-centered service behavior of medical professionals.

Furthermore, Zhang et al. (22) believed that gaining recognition and trust from patients and peers can increase medical professionals' motivation. Wu et al. (23) stated that good interpersonal relationships can stimulate medical students' self-efficacy. Thus, the literature shows that a positive social context, including policies and regulations, social relations, and patient support, can promote the self-efficacy of medical professionals, and in turn affect their patient-centered service behavior. Therefore, the following hypothesis was proposed.

**Hypothesis 3 (H**_**3**_**)**: Self-efficacy mediates the relationship between social context and patient-centered service behavior.

#### Moderating Effect of Achievement Motivation on the Relationship Between Social Context and Self-Efficacy of Medical Professionals

The concept of achievement motivation was introduced in 1938 by Murry (24), who defined it as the inherent psychological need of “overcoming obstacles, displaying talents, and striving to solve a difficult problem as soon as possible.” Atkinson (25) believed that achievement motivation shows opposite psychological effects in individuals: first, the pursuit of success motivation; and second, the avoidance of failure motivation. Employees with high achievement motivation are more likely to be attracted by job opportunities in the external social context, have high self-efficacy, and strive to pursue higher goals, thus being more likely to display a turnover tendency. Song et al. (26) conducted a study on standardized training residents in a Grade III Class A hospital in Xuzhou, and showed that achievement motivation played a negative role in predicting the level of job burnout. The results from this study indicated that standardized training residents with high achievement motivation could maintain a positive attitude at work, have a strong sense of self-efficacy, overcome emotional exhaustion, and treat patients with enthusiasm. Therefore, achievement motivation may regulate the influence of external social context on medical professionals' self-efficacy. Thus, the following hypothesis was formulated.

**Hypothesis 4 (H**_**4**_**):** Medical professionals' achievement motivation will have a moderating effect on the relationship between social context and self-efficacy.

## Methods

### Participants and Data Collection

The participants are medical professionals, including doctors, nurses, and medical technicians from different levels of public hospitals in Hangzhou city. It is an economically developed municipality located on the southeast coast of China, with a per capita GDP of USD 9, 696 in 2020. There are over 20 tertiary public hospitals, over 30 secondary public hospitals, and over 60 community health centers in this city. Using stratified random sampling method, 6 Grade III hospitals, 6 Grade II hospitals, and 15 community health service centers were randomly selected from the different levels of medical institutions in Hangzhou. Approximately 200, 50, and 20 professionals were selected from each Grade III hospital, Grade II hospital, and community health service center, respectively, according to the scale of the institutions. The inclusion criteria were (1) staff of the survey hospital and (2) experience of 6 months or more in their current position. Exclusion criteria were (1) not on duty during the survey period and (2) interns and foreign trainees.

This cross-sectional study was conducted by 2 research fellows and 5 graduates having adequate experience in on-site survey from July 1 to September 30, 2019. Before data collection, the ethics committee of Hangzhou Normal University reviewed and approved the research plan, informed consent document, as well as the questionnaire. Besides, the investigators were trained to apply the same standards and methods; face-to-face interview was used during data collection, when the purpose and meaning of this study were briefly introduced to subjects in order to get their consent, and necessary explanations were given; after the questionnaires were filled out, the investigators inspected them and checked with subjects regarding the questionnaires that did not meet the requirement. A total of 1,800 medical professionals were surveyed, and 1,612 valid questionnaires were recovered (recovery rate = 89.56%). The characteristics of the final 1,612 participants are listed in Table 1.

**Table 1:** Descriptive statistics of the sample (*N* = 1,612).

**Characteristic**	**Category**	**Frequency (*N*)**	**Composition ratio (%)**	**Characteristic**	**Category**	**Frequency (*N*)**	**Composition ratio (%)**
Gender	Male	419	26.0	Work experience (years)	<1	183	11.4
	Female	1,193	74.0		1–5	372	23.1
Marital status	Married	1,154	71.6		5–10	402	24.9
	Unmarried	426	26.4		10–15	287	17.8
	Divorced	21	1.3		15–20	137	8.5
	Other	11	0.6		≥20	231	14.3
Age (years)	<25	229	14.2	Staffing status	Permanent staff	1,199	74.4
	25–35	712	44.2		Non-permanent employment	403	25.6
	35–45	489	30.3	Average daily working hours (h)	<8	277	17.2
	≥45	182	11.3		8–10	1,175	72.9
Academic degree	College and below	221	13.7		10–12	128	7.9
	Undergraduate	1,142	70.8		≥12	32	2.0
	Masters or above	249	15.4	Average monthly income (yuan)	≤3,000	94	5.8
Professional title	To be assessed	177	11.0		3,001–5,000	320	19.9
	Primary title	602	37.3		5,001–7,000	581	36.0
	Middle title	556	34.5		7,001–10,000	459	28.5
	Vice-senior title	211	13.1		>10,000	158	9.8
	Senior title	66	4.1	Hospital level	Grade III	1,179	73.1
Job position	Doctor	591	36.7		Grade II	204	12.7
	Nurse	744	46.2		Community health service center	229	14.2
	Medical technician	256	15.9	Patient-centered communication skills training	Yes	958	59.4
	Other	21	1.3		No	654	40.6
Department	Internal medicine	501	31.1	Familiarity with patient-centered services	Very unfamiliar	56	3.5
	Surgery	125	7.8		Not very familiar	132	8.2
	Gynecology and obstetrics	96	6.0		General	639	39.6
	Pediatrics	64	4.0		Familiar	653	40.5
	Medical technology	230	14.3		Very familiar	132	8.2
	Emergency	70	4.3	Average diagnosis and treatment time (outpatient doctor only, min)	<5	71	4.4
	Ophthalmology and otorhinolaryngology	27	1.7		5–10	285	17.7
	Psychiatry	105	6.5		10–15	107	6.6
	Other	394	24.4		15–20	53	3.3
Whether a teaching hospital	Yes	1,259	78.1		≥20	49	3.0
	No	353	21.9				

### Measurement

A questionnaire was developed for this study using the following steps: (1) Items were screened through literature review; (2) Items were modified by two rounds of experts consultation; and (3) the questionnaire was further modified and verified by pre-survey. Internal consistency (Cronbach's α coefficient) and composite reliability (CR) were used to evaluate the reliability of the questionnaire, and the validity of the measurement was evaluated using content and aggregation validity. The questionnaire included the following sections: sociodemographic information questions, a self-efficacy measure, an achievement motivation measure, a social context measure, and a measure of medical professionals' patient-centered service.

The self-efficacy questionnaire (27) included six items, mainly assessing the participants' confidence in communication and interaction with patients (e.g., “I have a strong ability to establish mutually trusting relationships with patients”). Each item was measured on a five-point Likert scale ranging from 5 (*strongly agree*) to 1 (*strongly disagree*); with higher scores indicated higher self-efficacy. The Cronbach's α coefficients and CR values of the self-efficacy questionnaire were 0.920 and 0.701, factor loading was from 0.764 to 0.889 the AVE value was 0.701, the correlation coefficient between each item and the overall score was 0.745~0.853 (*p* < 0.001), indicating that the measures had good reliability and validity (Table 2).

**Table 2:** Measurement items and results of reliability and validity analysis of the questionnaire (*N* = 1,612).

**Construct**	**Dimension**	**Measurement items**	**Load^**a**^**	**Cronbach's α**	**Correlation coefficient**	**AVE**	**CR**	**Overall α value**
Self-efficacy	Self-efficacy	A1 I have a strong ability to establish mutually trusting relationships with patients	0.837	0.920	0.834^**^	0.701	0.701	0.920
		A2 I can usually be more friendly to patients	0.764		0.750^**^			
		A3 I have a strong ability to detect patients' nonverbal cues/behaviors	0.838		0.853^**^			
		A4 I have the ability to ask patients some specific questions at the right time	0.889		0.824^**^			
		A5 I can explain medical terms in plain language	0.887		0.750^**^			
		A6 I ask patients about their sensitivity and personal issues, and the patients are willing to communicate with me	0.851		0.745^**^			
Achievement motivation	Pursue success	When I get patients' affirmation, I feel very happy	0.859	0.920	0.588^**^	0.778	0.934	0.860
		I strive to provide personalized medical services for patients	0.892		0.528^**^			
		I meet the reasonable needs of patients and try my best to find ways to solve their difficulties	0.915		0.538^**^			
		I like to finish the tasks assigned to me as soon as possible	0.862		0.619^**^			
	Avoid failure	When the effect of diagnosis, treatment, examination, or care of patients is unclear, I feel uneasy	0.821	0.851	0.586^**^	0.670	0.890	
		I feel anxious when I cannot reach a consensus with patients on diagnosis, treatment, examination, or care decisions	0.876		0.566^**^			
		I hate dealing with medical accidents, disputes, etc.	0.709		0.576^**^			
		When I cannot immediately understand the patient's questions, I feel anxious and uneasy	0.859		0.616^**^			
Patient-centered service	Effective communication	In most cases, I provide patients with detailed information about the diagnosis, treatment, examination or the care they need	0.685	0.857	0.789^**^	0.433	0.694	0.967
		In most cases, I give patients sufficient time to consult	0.720		0.780^**^			
		In most cases, I communicate with patients in a mild tone	0.558		0.818^**^			
	Patient focused	In most cases, I can listen patiently while a patient is speaking	0.632	0.905	0.866^**^	0.434	0.691	
		In most cases, I am concerned about the extent to which patients understand their own condition/prognosis	0.786		0.824^**^			
		In most cases, I understand the patient's preferences and needs	0.533		0.866^**^			
	Empathy	In most cases, I value the protection of patients' privacy	0.802	0.933	0.798^**^	0.457	0.805	
		In most cases, I am concerned about patients' expectations of medical outcomes	0.753		0.805^**^			
		In most cases, I can sympathize with patients' bad mood	0.594		0.865^**^			
		In most cases, I can put myself in the patient's shoes	0.612		0.878^**^			
		In most cases, I can provide confidence and security to patients	0.588		0.878^**^			
	Patient engagement	In most cases, I can reach a consensus with the patients and deal with conflicts together	0.695	0.948	0.892^**^	0.575	0.871	
		In most cases, I encourage patients to participate in discussions and decisions about diagnosis, treatment, examination, or care	0.780		0.825^**^			
		In most cases, I can arouse the patients' attention to the disease and give timely guidance	0.774		0.887^**^			
		In most cases, I allow patients to ask questions and express their views when asking about their illness	0.764		0.876^**^			
		In most cases, I will try to understand why patients do not actively participate in the service process	0.776		0.821^**^			
Social context	Policies and regulations	The government provides the necessary financial support to ensure patients' access to basic medical services	0.611	0.939	0.552^**^	0.671	0.924	0.962
		The medical ethics supervision system is sound	0.778		0.651^**^			
		The hospital should establish a clear working system and post responsibility requirements	0.872		0.691^**^			
		The hospital has a humanized service system	0.888		0.688^**^			
		The hospital has established a perfect medical price transparency publicity system	0.865		0.673^**^			
		The hospital strictly implements the Basic Standard for Writing Medical Records and monitors and manages the quality of medical records throughout the process	0.867		0.673^**^			
	Social relations	Social respect and understanding of medical professionals promote my patient-centered service behavior	0.947	0.942	0.950^**^	0.897	0.963	
		A harmonious and stable doctor–patient relationship promotes my patient-centered service behavior	0.958		0.947^**^			
		Objective and fair media coverage of the medical industry promotes my patient-centered service behavior	0.936		0.947^**^			
	Patient support	Patients' objective and reasonable expectation of medical results promotes my patient-centered service behavior	0.888	0.928	0.897^**^	0.766	0.951	
		Patients with high health literacy level promote my patient-centered service behavior	0.907		0.783^**^			
		Patients with better economic conditions promote my patient-centered service behavior	0.636		0.500^**^			
		Patients' active cooperation with medical professionals in treatment promotes my patient-centered service behavior	0.926		0.814^**^			
		Patients' ability to actively adjust their psychological state promotes my patient-centered service behavior	0.937		0.795^**^			
		Patients with strong communication skills promote my patient-centered service behavior	0.918		0.762^**^			

a *all load values are significant at the 0.001 level; CR, composite reliability; AVE, average variance extraction*.

** *p < 0.01, two-tailed test*.

The achievement motivation questionnaire (28) included two dimensions related to pursuing success and avoiding failure, with five items in each dimension (e.g., “I strive to provide personalized medical service for patients”). Each item was measured on a five-point Likert scale ranging from 5 (*strongly agree*) to 1 (*strongly disagree*); and items in the avoiding failure dimension were reverse scored. Higher scores indicated stronger achievement motivation. The Cronbach's α coefficients of the total scale and dimensions were above 0.800, the CR values were 0.934 and 0.890, factor loading was from 0.709 to 0.915; the AVE values were 0.778 and 0.670, the correlation coefficient between each item and the overall score was 0.528~0.619 (*p* < 0.001), indicating that the measures had good reliability and validity (Table 2).

The self-developed social context questionnaire (29–31) included three dimensions: policies and regulations (6 items), social relations (3 items), and patient support (6 items). For example, the policies and regulations dimension included items such as “social respect and understanding for medical professionals will drive me to engage in patient-centered service.” The five-point Likert-type rating scale ranged from 5 (*strongly agree*) to 1 (*strongly disagree*). Higher scores indicated a positive social environment conducive to a medical staff's patient-centered medical service. The Cronbach's α coefficients of the total scale and dimensions were above 0.800, the CR values of dimensions were from 0.924 to 0.963, factor loading was from 0.611 to 0.958; the AVE values between 0.671 to 0.897, the correlation coefficient between each item and the overall score (0.500–0.950) (*p* < 0.001), indicating that the measures had good reliability and validity (Table 2).

Medical professionals' patient-centered service was evaluated using the Provider–Patient Relationship Questionnaire by Gremigni et al. (32), which includes four dimensions: effective communication (4 items), patient care (4 items), empathy (4 items), and patient participation (4 items). Responses were measured on a five-point Likert scale ranging from 5 (*never*) to 1 (*always*); higher scores indicated better patient-centered services. The Cronbach's α coefficients of the total scale and dimensions were above 0.800, the CR values of dimensions were from 0.691 to 0.871, factor loading was from 0.533 to 0.802; the AVE values between 0.433 to 0.575, the correlation coefficient between each item and the overall score (0.780–0.892) (*p* < 0.001), indicating that the measures had good reliability and validity (Table 2).

### Ethical Considerations

The purpose of the study and procedures for data collection were explained to the participants prior to the data collection. The participants were informed that participation in the study was entirely voluntary and that their data would be anonymized using pseudonyms and would only be used for research purposes. Thereafter, written informed consent was obtained from the participants. Ethical approval for the study (approval number: 2021-1146) was obtained from the Institutional Review Board of Hangzhou Normal University. Data were collected via an anonymous field survey.

### Statistical Analysis

Initially, normality, outliers, and multicollinearity were evaluated. Normality was assessed using coefficients of skewness (sk) and kurtosis (ku). Values fell within the acceptable ranges of −0.863–0.362 for sk and 0.576–3.092 for ku. The existence of outliers was identified by Cook's distance. The maximum Cook's distance was <0.5 (0.201), which indicated there were no outliers in these data. Multicollinearity was tested by the tolerance rate and variance inflation factor (VIF). The findings showed no tolerance rate <0.10 or VIF > 10. All the tolerance values were >0.211 and the VIF was <4.746, which indicated no multicollinearity.

SPSS 26.0 and Amos 22.0 (Armonk, NY.IBM.Corp) were used for statistical analyses. Internal consistency (Cronbach's α coefficient) and composite reliability (CR) were used to evaluate the reliability of the questionnaire. The validity of the measurement was evaluated using content and aggregation validity. Spearman's correlation analysis was used to calculate the correlations among the variables and their dimensions. The driving path from social context and self-efficacy to patient-centered service behavior was estimated and verified using the maximum-likelihood method in the structural equation model (SEM) analysis. Structural equation model is a multivariate linear statistical modeling method that includes model construction, model correction, and model interpretation. The variables in the model can be categorized into latent variables (indirectly measured with the help of observed variables) and observed variables (directly measured). The model not only measures the reliability and validity of the scale but also determines the influencing factors and clarifies the relationships among the influencing factors, which is an incomparable advantage over traditional regression analysis methods (33). Therefore, to explore the driving mechanism of medical professionals' patient-centered service behavior, Amos 22.0, was used to construct the initial hypothesis model and the maximum-likelihood method was used to estimate the initial model. Finally, using hierarchical linear regression analysis, this study included interactive items to test whether achievement motivation had a moderating effect in the relationship between social context andself-efficacy.

## Results

### Descriptive Statistics and Correlation Analysis

Self-efficacy, achievement motivation, policies and regulations, social relations, and patient support had significant positive correlations with effective patient-centered communication, patient care, empathy, and patient participation; indicating that further testing with the SEM and a regression model is suitable (Table 3).

**Table 3:** Mean, standard deviation, and correlation coefficient of each variable (*N* = 1,612).

**Variable**	**1**	**2**	**3**	**4**	**5**	**6**	**7**	**8**	**9**
1. Self-efficacy									
2. Achievement motivation	0.217^***^								
3. Policies and regulations	0.494^***^	0.224^***^							
4. Social relations	0.456^***^	0.232^***^	0.720^***^						
5. Patient support	0.476^***^	0.171^***^	0.680^***^	0.822^***^					
6. Effective communication	0.522^***^	0.241^***^	0.615^***^	0.592^***^	0.597^***^				
7.Patients focused	0.553^***^	0.244^***^	0.596^***^	0.568^***^	0.585^***^	0.820^***^			
8. Empathy	0.555^***^	0.293^***^	0.642^***^	0.600^***^	0.606^***^	0.801^***^	0.837^***^		
9.Patients engagement	0.565^***^	0.240^***^	0.600^***^	0.568^***^	0.602^***^	0.743^***^	0.796^***^	0.853^***^	
Mean value	23.431	26.530	20.510	12.426	24.419	12.450	12.373	21.060	20.593
Standard deviation	3.828	3.099	3.239	2.153	4.0338	1.883	1.883	3.021	3.198

*** *p < 0.001, two-tailed test*.

### Structural Equation Model Analysis

#### Construction and Fit of the Model

**Figure 2:**
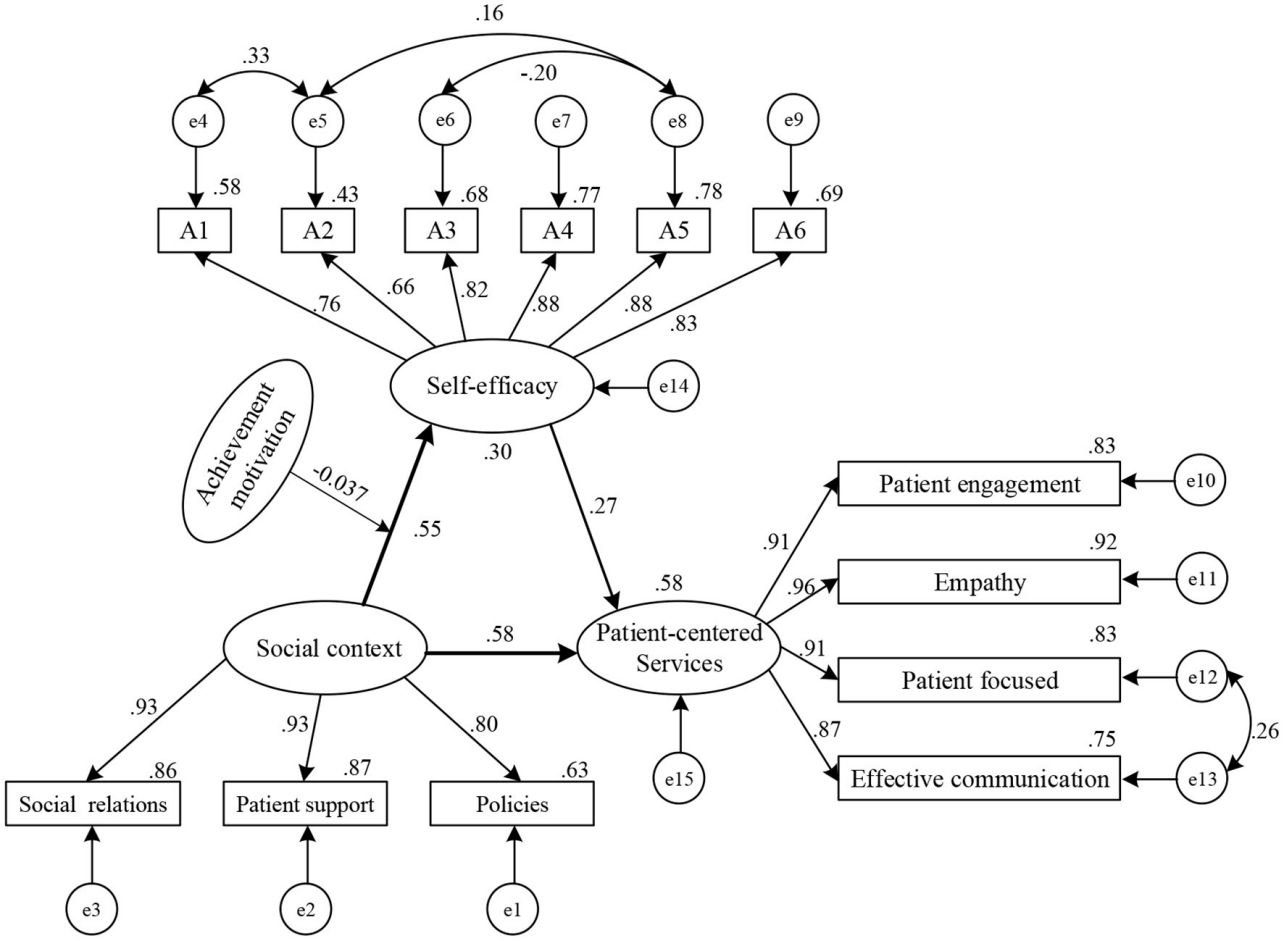
Model diagram of driving mechanism of medical professionals' patient-centered service behavior.

In this study, the social context and self-efficacy were taken as exogenous latent variables, and patient-centered service behavior was divided into endogenous latent variables to construct a SEM. The initial model was estimated using the maximum-likelihood method. The results of the model fitting parameters showed that the *p*-value of each path was <0.05, which was reserved, and the model was modified using the correction index. Four residual paths, [e4–e5], [e6–e8], [e5–e8], and [e12–e13] were added. The final model is shown in Figure 2; each fitness index had a good fit; see Table 4 for details.

**Table 4:** Fitting results of the structural equation model.

**Fitting index**	**Fitting standard**	**Initial model**	**Modified model**
Chi-square freedom ratio χ^2^/*df*	1 < χ^2^/*df* <3 Good	13.245	9.252
Root mean square error of approximation RMSEA (90%CI)	<0.08 Acceptable	0.084 (0.079–0.089)	0.078 (0.064–0.074)
Goodness of fit index, GFI	>0.90 Good	0.929	0.954
Adjusted goodness of fit index, AGFI	>0.90 Good	0.896	0.928
Normed fit index, NFI	>0.90 Good	0.961	0.975
Confirmatory fit index, CFI	>0.90 Good	0.964	0.977
Bentler and Bonett's non-normed fit index, TLI	>0.90 Good	0.955	0.969

#### Effect Relationship and Hypothesis Verification

Social context had both direct and indirect effects on patient-centered service behavior; the direct and indirect effect values were 0.578 and 0.149, respectively (Table 5). The 95% confidence intervals in the bias-corrected and percentile columns belonging to the partial mediation effect did not include 0, supporting H_1_ and H_3_. Self-efficacy had only a direct impact on patient-centered service behavior, with an effect value of 0.269. The 95% confidence interval did not include 0; thus, H_2_ was supported.

**Table 5:** Bootstrap confidence interval estimation results.

**Variable relationship**	**Effect type**	**Effect value**	**Percentile**		**Bias-corrected**		**Supported hypothesis**
Social context → Patient-centered service			Lower limit	Upper limit	Lower limit	Upper limit	H_1_, H_3_
	Overall effect	0.727	0.674	0.769	0.674	0.766	
	Direct effect	0.578	0.517	0.636	0.516	0.635	
	Indirect effect	0.149	0.111	0.185	0.110	0.185	
Self-efficacy → Patient-centered service	Overall effect	0.269	0.207	0.333	0.206	0.331	H_2_
	Direct effect	0.269	0.207	0.333	0.206	0.331	
	Indirect effect	\	\	\	\	\	

### Hierarchical Linear Regression Analysis

To test the moderating effect of achievement motivation between social context and self-efficacy, a hierarchical linear regression analysis was conducted. Before carrying out the test, to avoid multicollinearity between the variables, the independent variables (social context), dependent variables (self-efficacy), and moderating variables (achievement motivation) were centralized, and the interactive terms of social context and achievement motivation were constructed. Three models were constructed. The maximum variance expansion factor was 7.025, which was significantly lower than 10. No issue of multicollinearity was detected, and the results were reliable.

Model 1 introduced statistically significant control variables in the univariate analysis (Table 6). The results showed that gender had a significant effect on the achievement motivation of medical professionals, with female medical professionals demonstrating higher achievement motivation than their male counterparts (β = 0.057, *p* < 0.05). The higher the medical professionals' familiarity with patient-centered services, the higher was their self-efficacy (β = 0.150, *p* < 0.001). Model 2 introduced independent variables (social context) and regulatory variables (achievement motivation) based on Model 1. The results showed that the main effects of social context and achievement motivation on self-efficacy were positive (β = 0.691, *p* < 0.001; 0.084, *p* < 0.001), indicating that social context and achievement motivation had positive effects on self-efficacy. On the basis of Model 2, Model 3 introduced the interactive items of independent variables and regulatory variables (social context^*^achievement motivation). The results showed that achievement motivation had a negative regulatory effect on the relationship between social context and self-efficacy (β = −0.037, *p* < 0.001).

**Table 6:** Moderating effect of achievement motivation of medical professionals.

**Variable**	**Self-efficacy**		
	**Model 1**	**Model 2**	**Model 3**
**Gender**			
Female (reference group)			
Male	0.057^**^	0.026	0.025
**Age (years)**			
≤25 (reference group)			
25–35	0.034	0.015	0.017
35–45	0.072	0.069^*^	0.070^*^
≥45	0.026	0.064^*^	0.064^*^
**Professional title**			
**To be assessed (reference group)**			
Primary title	−0.026	0.000	−0.003
Middle title	0.002	0.025	0.022
Vice-senior title	0.024	0.035	0.033
Senior title	0.02	0.023	0.022
**Job position**			
**Doctor (reference group)**			
Medical technician	−0.102^**^	−0.073^***^	−0.072^***^
Nurse	−0.057^**^	−0.089^***^	−0.088^***^
**Working experience (years)**			
≤1 (reference group)
1–5	−0.091^**^	−0.021	−0.021
6–10	−0.072	0.001	0.000
11–15	−0.089^*^	−0.005	−0.006
16–20	−0.052	−0.016	−0.017
>20	−0.061	0.003	0.002
**Staffing status**			
**Permanent staff (reference group)**			
Non-permanent employment	0.016	0.022	0.021
Trainee/visiting scholar	−0.023	0.019	0.018
**Average monthly personal income (yuan)**			
≤3,000			
3,001–5,000	0.025	0.048	0.049
5,001–7,000	0.062	0.067	0.065
7,001–10,000	0.089	0.053	0.052
>10,000	0.067	0.011	0.010
**Communication skills training**			
Yes (reference group)			
No	−0.073^**^	0.001	−0.001
Familiarity with patient-centered services	0.150^***^	0.057^***^	0.059^***^
Social context		0.691^***^	0.678^***^
Achievement motivation		0.084^***^	0.089^***^
Social context*achievement motivation			−0.037^**^
R2	0.064	0.55	0.552
Adjusted R2	0.051	0.544	0.545
ΔR2	0.064	0.487	0.001
Variance ratio	5.039^***^	83.436^***^	80.572^***^
ΔF	5.039	922.414	4.580
VIF_max_	6.669	7.023	7.025

* *p < 0.05*,

** *p < 0.01*,

*** *p < 0.001; VIF_max_, maximum variance expansion factor*.

**Figure 3:**
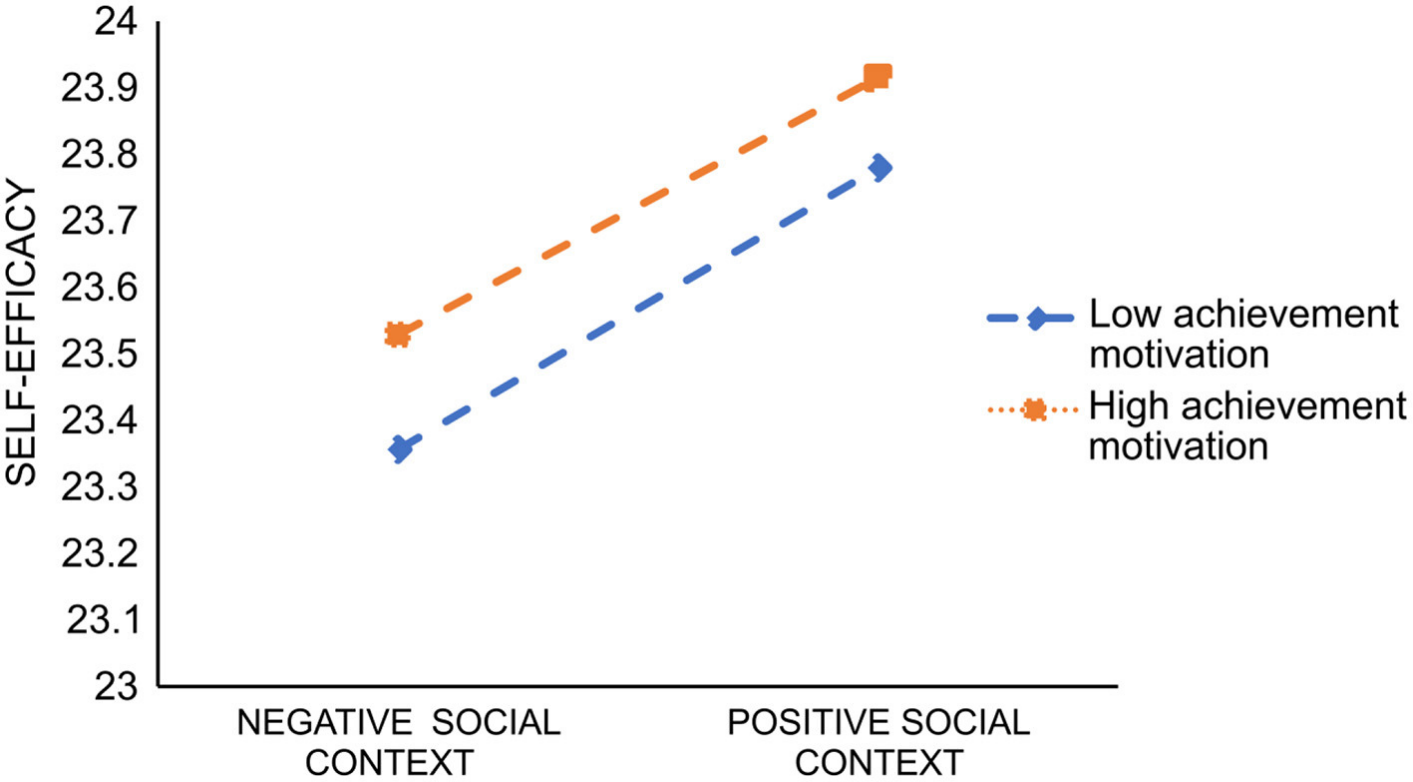
Moderating effect diagram of achievement motivation.

According to the research results, we plotted the moderating effect chart of achievement motivation. With improvement in social context, medical professionals' self-efficacy showed an upward trend, regardless of achievement motivation; thus, social context had a good universality in promoting medical professionals' self-efficacy (Figure 3). Additionally, the result reflects that with high achievement motivation, the promotion effect of social context on medical professionals' self-efficacy is higher.

## Discussion

### Theoretical Significance

There were three new discoveries in this study. First, social context influences the patient-centered service behavior of medical professionals directly as well as indirectly through self-efficacy. This finding supports the results of previous studies showing that sound rules and regulations, and good social networks can clarify the responsibilities and obligations to medical professionals, thus enabling them to put a sincere effort toward their work and encouraging appropriate behaviors (34–36). The establishment of corresponding supporting policies by the government, adequate responsibilities and service systems in hospitals, and stable interpersonal relationships can promote the patient-centered service behavior of medical professionals. Further, some studies have shown that the lack of communication management standards in medical institutions, poor interpersonal relations with colleagues, and disregard for patients' requests hinder doctor–patient communication (37). Patients' lack of trust in medical professionals leads to poor communication between doctors and patients, such that medical professionals are unable to accurately perceive and understand the patients' inner worlds (38). Thus, patients' trust and support can promote medical professionals to pay attention to them, promote empathy, and encourage patients to actively participate in medical decision-making. Therefore, it is necessary to report medical issues fairly and justly, stimulate medical professionals' patient-centered empathy, improve the health literacy level and participation ability of patients and their families, and create a good atmosphere for both doctors and patients.

Second, self-efficacy has a direct positive effect on the patient-centered service behavior of medical professionals. The stronger the self-efficacy, the stronger is their confidence in effective communication and interpersonal communication, and the better they are at being able to adapt to changes in the environment, manage themselves, deal with practical clinical problems, and strengthen their service ability (39). Further, the external social context can indirectly influence the patient-centered service behavior of medical professionals through self-efficacy, which is in line with Zhang's viewpoint (40). Studies have shown that the support of the organization and external social context can improve the insight and emergency response of medical professionals, enhance doctors' confidence in their communication abilities, promote the effectiveness of doctor–patient communication, and the initiative of service (41). A supportive environment can facilitate the enhancement of medical professionals' self-efficacy (42). Patients' understanding and support for medical professionals can meet the latter's needs for personal value and social respect, enhancing their professional confidence and improving their enthusiasm for work and professional dedication (43).

Third, achievement motivation plays a moderating role in the relationship between social context and self-efficacy. The higher the achievement motivation of medical professionals, the stronger is the positive effect of social context on their self-efficacy. In the context of high achievement motivation, with gradual improvement in the social context, the self-efficacy of medical professionals is promoted, and the promotion effect is more significant than that of low achievement motivation. This is similar to the findings reported by Wei (44). Additionally, a study by Li (45) showed that people with high achievement motivation pursue self-improvement, tend to seize opportunities in the external social context, make effective use of resources, and have higher self-efficacy throughout the process. Social context is important to medical personnel governed by achievement motivation, perhaps because they cherish this social context and are willing to make positive contributions, resulting in stronger self-efficacy. Therefore, it is necessary to create a favorable social context and help medical professionals set high achievement goals, manage difficult tasks, and enhance their achievement motivation, to promote their self-efficacy and ultimately their patient-centered service behavior.

These findings are in line with motivation theory as both, internal and external motivation drive individual attitudes or behaviors.

### Limitations and Future Prospects

Some of the possible shortcomings of this study are as follows. First, a bias in the research object selection may be present; participants may have chosen to participate in this study because they were focused on providing patient-centered service. Second, as a self-report measure was used for data collection, the possibility of response bias cannot be ruled out. Third, only the medical professionals' perspectives were considered in this study; the patients' perspectives were excluded. Fourth, the study could not capture the causal effect of changes over time due to the cross-sectional nature of the data. Therefore, longitudinal research could help unravel the direction of causality in the relationship between the internal and external motivation effects, self-efficacy, and social context on the patient-centered service behavior of medical professionals. Finally, as all participants were sampled from hospitals in Hangzhou, the results may not be representative of the influencing factors and mechanisms of patient-centered services in other regions. However, all hospitals were public owned, which accounted for the overwhelming majority of the Chinese healthcare providers, funded by the government and with a similar structure. Therefore, future research should include objective evaluation indicators, survey both doctors as well as patients to further explore the influencing factors of patient-centered services, and consider collecting data from other regions.

### Practice Implications

This study demonstrates that social context and self-efficacy affect the patient-centered service behavior of medical professionals. This finding is significant for the transformation and improvement of hospital management.

On one hand, the government, health administrative department and hospitals can encourage patient-centered behaviors of health providers by improving the circumstances. For instance, they should invest more into the healthcare industry through relevant policies, which increase the income of health workers, and gradually stop hospitals from gaining revenues from excessive drug sales. Regarding social relations, they should urge the public to respect health workers and appreciate their work. Regarding the aspect of patient support, they should strengthen the trust of patients with health professionals and cultivate a harmonious relationship between doctors and patients. On the other hand, they can work on psychological elements like stimulating self-efficacy and motivation of health professionals. For example, health workers should be encouraged to participate in seminars or professional training, thereby improving their expertise and ethics. Positive motivation should be developed in health workers, and their awareness of effective communication and confidence in interpersonal association should be increased with training in communication skills, thereby boosting their self-efficacy. The ability of listening to patients patiently and identifying their physical and psychological needs precisely should be promoted with training in empathy.

## Conclusion

The external social context, such as policies and regulations, social relations, and patient support, can directly influence the patient-centered service behavior of medical professionals as well as influencing it indirectly by affecting medical professionals' self-efficacy. With gradual improvements in the social context, the self-efficacy of medical professionals can be increased. Especially among medical professionals with high achievement motivation, the social context has a more significant role in promoting self-efficacy.

## Data Availability Statement

The datasets presented in this study can be found in online repositories. The names of the repository/repositories and accession number(s) can be found at: https://www.wjx.cn/wjx/design/previewmobile.aspx?activity=44059765&s=1.

## Ethics Statement

The studies involving human participants were reviewed and approved by Institutional Review Board of Hanghzou Normal University. The patients/participants provided their written informed consent to participate in this study.

## Author Contributions

XH: conceptualization, methodology, and software. LH: visualization and investigation. CW: formal analysis and writing—original draft preparation. YG: software and validation. SZ: supervision. XW: writing—review and editing. All authors approved the final version of the manuscript.

## Funding

This work was supported by the National Natural Science Foundation of China Project (Grant No. 71974050) and the Soft Science Research Program of Zhejiang Provincial Science and Technology Plan (Grant No. 2021C35012).

## Conflict of Interest

The authors declare that the research was conducted in the absence of any commercial or financial relationships that could be construed as a potential conflict of interest.

## Publisher's Note

All claims expressed in this article are solely those of the authors and do not necessarily represent those of their affiliated organizations, or those of the publisher, the editors and the reviewers. Any product that may be evaluated in this article, or claim that may be made by its manufacturer, is not guaranteed or endorsed by the publisher.
